# Selection for long lifespan in men: benefits of grandfathering?

**DOI:** 10.1098/rspb.2007.0688

**Published:** 2007-07-24

**Authors:** M Lahdenperä, A.F Russell, V Lummaa

**Affiliations:** 1 Section of Ecology, Department of Biology, University of Turku20014 Turku, Finland; 2 Department of Animal and Plant Sciences, University of SheffieldSheffield S10 2TN, UK

**Keywords:** ageing, cooperative breeding, *Homo*, life history evolution, longevity, senescence

## Abstract

Life-history theory suggests that individuals should live until their reproductive potential declines, and the lifespan of human men is consistent with this idea. However, because women can live long after menopause and this prolonged post-reproductive life can be explained, in part, by the fitness enhancing effects of grandmothering, an alternative hypothesis is that male lifespan is influenced by the potential to gain fitness through grandfathering. Here we investigate whether men, who could not gain fitness through reproduction after their wife's menopause (i.e. married only once), enhanced their fitness through grandfathering in historical Finns. Father presence was associated with reductions in offspring age at first reproduction and birth intervals, but generally not increases in reproductive tenure lengths. Father presence had little influence on offspring lifetime fecundity and no influence on offspring lifetime reproductive success. Overall, in contrast to our results for women in the same population, men do not gain extra fitness (i.e. more grandchildren) through grandfathering. Our results suggest that if evidence for a ‘grandfather’ hypothesis is lacking in a monogamous society, then its general importance in shaping male lifespan during our more promiscuous evolutionary past is likely to be negligible.

## 1. Introduction

Life-history theory suggests that selection should have favoured individuals who maximize their fitness (Stearns 1992; Roff 2001). In most species, this is achieved by breeding throughout adult life. However, in cooperatively breeding species, individuals can accrue inclusive fitness by helping kin (Hamilton 1964; Griffin & West 2003; Lee 2003). Although lifespan can be unusually long in such cooperative species (Keller & Genoud 1997), few show the prolonged post-reproductive life characteristic of human females. Regardless of the actual evolutionary origin of menopause and/or prolonged post-reproductive lifespan (Lahdenperä *et al*. 2004*a*), it is now undeniable that women can gain at least some fitness following menopause through helping their offspring and grandmothering (Hawkes *et al*. 1998; Sear *et al*. 2002, 2003; Voland & Beise 2002; Jamison *et al*. 2002; Lahdenperä *et al*. 2004*b*; Ragsdale 2004; Tymicki 2004; Beise 2005; Gibson & Mace 2005; Kemkes-Grottenthaler 2005). Consequently, lifespan in women must, at least in part, be influenced by the benefits of post-reproductive helping.

The question then is why do men live almost as long as women? As is the case for women, the long lifespan observed in men is not just a modern day artefact; data from modern hunter–gatherers suggest that a large proportion of the adulthood population may be over the age of 50 (Hawkes & Blurton Jones 2005). The function of long lifespan in men has received considerably less attention, presumably because the traditional explanation has been that lifespan and reproductive capacity are linked together in men, as is the case in most animals, and men simply live until they begin to senescence reproductively (Williams 1957). Evidence from polygynous societies in which men are able to father offspring until death upholds this traditional view (Ratcliffe *et al*. 2000). However, by contrast, in polyandrous and monogamous mating societies, men have little ability to gain direct fitness once their wife reaches menopause (Paget & Timaeus 1994). In such societies, there should be little selection for a man living beyond his wife's reproductive years unless men, like women, are able to gain inclusive fitness benefits from remaining alive. Under a ‘grandfather hypothesis’, men might benefit by surviving to a similar age as women because they, like women, are able to gain fitness by improving the survival and reproductive success of their offspring. Although such an idea is seldom considered, there is some evidence to suggest that fathers could benefit their offspring by remaining alive beyond the age when they cease reproduction (table 1). For example, in contemporary Gambians, the presence of a father increases the probability that his sons will reproduce (Sear *et al*. 2003) and reduces his daughters' age at first reproduction (Allal *et al*. 2004). Moreover, father presence was found to be positively associated with offspring reproductive success in historical/contemporary Poles (Tymicki 2004). However, these types of findings are by no means universal (table 1), and studies to date have failed to investigate the effect of father presence on all key life-history traits and fitness measures of offspring to determine the potential selective benefits of grandfathering as a whole.

The aim of our study is to test the grandfather hypothesis fully using a multigenerational, individual-based dataset of pre-industrial Finns (Luther 1993). Our demographic data have at least five benefits for addressing our aim. First, they were collated from church registers maintained by local clergymen who were obliged by law to submit to the state accurate records of the survival and reproductive histories of all individuals in their parish area. Second, the study period coincides with periods of natural fertility and mortality, and ends before health care and more liberal economics improved standards of living in Finland (Soininen 1974). Third, the data come from three geographically distinct populations, from two ecotypes (archipelago and inland; Lummaa *et al*. 1998), and include the social class of each man (low, middle, high), allowing us to estimate the generality of our findings and control for differences in ecology and standards of living (Lahdenperä *et al*. 2004*b*). Fourth, we have shown previously using the same dataset that mother presence is associated with a substantial reproductive benefit for her offspring and that mothers gain significant fitness (in the form of increased numbers of grandoffspring) by surviving beyond menopause (Lahdenperä *et al*. 2004*b*). Finally, our data come from a strictly monogamous society where remarriage was only permitted after the death of a spouse (Sundin 1992). This is beneficial for the purposes of our study since monogamy and paternal investment are linked and we would expect the potential benefits of grandfathering to be greatest in a monogamous society (Marlowe 2000).

Specifically, we investigate the effect of father presence on (i) key life-history traits of his sons and daughters (age at first reproduction, inter-birth intervals and reproductive tenure length) and (ii) correlates of their fitness (lifetime fecundity, offspring survival and lifetime reproductive success). We then test (iii) the relationship between male longevity and the total number of grandchildren born (i.e. evolutionary fitness). In all analyses, we restrict our data to only those men who married once and hence had to stop reproducing after the death of their wife and, where appropriate, we control for possible confounding effects of wife presence, birth cohort, ecology, socio-economic status, father age (even if dead), and offspring birth order and sex, as well as repeated measures of the father or grandfather.

## 2. Material and methods

The Finnish data were collected using historical church records. The Lutheran church has been obliged by law to submit accurate registers of all births, inter-parish movements, marriages and deaths all over the country since the seventeenth century (Luther 1993). The Finnish church registers are one of the most reliable sources of demographic data on historical humans (Luther 1993). Our data contain three generations of full reproductive history and survival details from three geographically distinct communities: two coastal parishes (Kustavi and Rymattylä) and one inland parish (Ikaalinen). The source of livelihood was farming, supplemented by fishing in coastal areas leading to greater predictability of food in coastal areas (Lummaa *et al*. 1998). In the analyses, the two coastal parishes were grouped together to allow contrasts between the two ecological areas, coastal versus mainland. Overall, we collated data from 361 men born during the years 1719–1839, as well as the complete life history of their 2,277 offspring (of whom 942 married and reproduced in the population) and the complete survival history of their 4683 grandchildren. Of these men, 265 married only once during their lifetime (born during the years 1719–1823) and were included in the analyses (having 1568 children of whom 968 survived to age 15, and 674 married and reproduced). Our sample sizes and data included here differ slightly from those of our previous study of grandmothers (Lahdenperä *et al*. 2004*b*), because we restricted men to those marrying once during their lifetime and male data were only collected for three of the five study parishes. The study era ended before industrialism and therefore before more liberal economics, birth control methods and higher standards of living improved survival (Soininen 1974). The occupation (for example tenant farmer, fisherman, landowner, servant) of each man was recorded at the time the children were born and allowed us to rank the socio-economic status (rich, average, poor) of each family (Lahdenperä *et al*. 2004*b*). Extramarital affairs were a strict taboo of the Lutheran church and punishable (Sundin 1992). The rate of extra-pair paternity in our study population was probably as low as the 1.7–3.3% suggested for modern populations with high paternity confidence, or at least substantially lower than the median worldwide extra-pair paternity rate of 9% (Anderson 2006).

Statistical analyses were conducted using SAS (SAS Institute, Inc., release 9.1, 2002–2003). In each analysis, selection of significant confounding terms (see below) was determined using backward elimination. Whether the father/grandfather was alive or dead was entered into each model last. Backward and forward selection processes gave qualitatively similar results. Satterwaite's formula (Littell *et al*. 1996) was used to approximate the denominator degrees of freedom of each fixed effect in mixed models. All two- and three-way interactions involving father presence/absence were tested, but only presented where significant.

### (a) Father presence and offspring life-history traits

Effects of father presence on offspring life-history traits were analysed using general linear mixed models (GLMMs), which allow both fixed and random terms to be fitted in the model, with random terms taking into consideration repeated measures of individuals (Schall 1991). Three underlying life-history traits were considered in three separate analyses, age at first reproduction, inter-birth interval and reproductive tenure length (years between first and last child from the same wife). All three have been shown to be key determinants of fitness in Finns (Helle *et al*. 2005; Pettay *et al*. 2005). In our three analyses, the response term was fitted to a normal error structure and father presence/absence was fitted as the main fixed effect. In the analyses of age at first reproduction and reproductive tenure length, father presence/absence was defined at the time each of their offspring reproduced for the first time. (Reproductive tenure length was not influenced by the number of years for which fathers were alive following offspring's start of first reproduction (GLMM, *F*_1,138_=0.09, *p*=0.77, *n*=220 offspring, 105 fathers (1–7 measures/father)). In the analysis of inter-birth intervals, father presence/absence was defined at the birth of each grand offspring.

**Figure 1:**
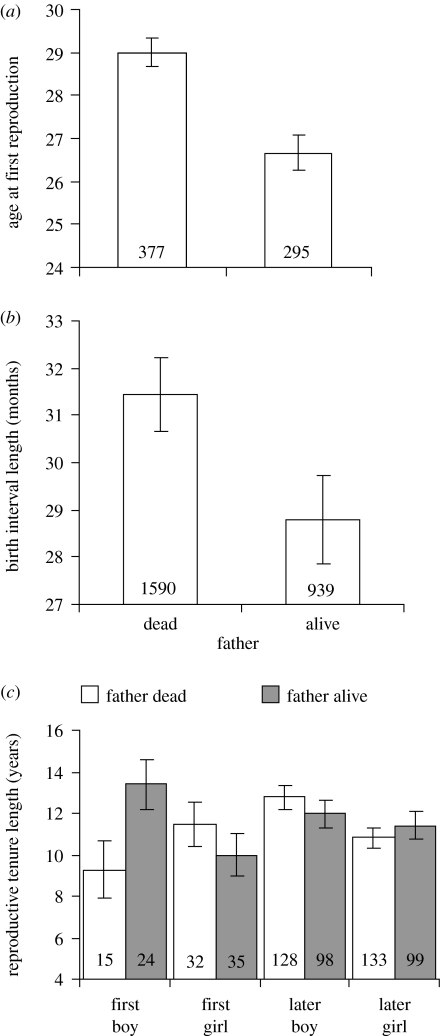
Father presence and correlates of offspring key life-history traits. A living father is associated with: (*a*) lower age at first reproduction of all of his offspring (GLMM: *F*_1,439_=27.65, *p*<0.0001); (*b*) reduced inter-birth intervals of all of his offspring (GLMM: *F*_1,926_=12.58, *p*=0.0004); and (*c*) increased reproductive tenure length of first-born son's only (GLMM: interaction: *F*_1,550_=6.91, *p*=0.0088). First boy, first-born son; first girl, first-born daughter; later boy, later-born son; later girl, later-born daughter. Graphs show predicted means (±1 s.e.) from GLMMs after controlling for significant effects outlined in the relevant section of the results and repeated measures of father (*p*_(a–c)_*=*0.0008,<0.0001, 0.04, respectively).

Father effects on age at first reproduction (figure 1*a*) were investigated for 672 offspring sired by 230 fathers (1–8 measures/father). The offspring were restricted to those who had their first child before age 45.5, which was the age when 99% had already started reproducing. Father effects on inter-birth intervals (figure 1*b*) were investigated using 2529 birth intervals by 560 offspring (1–15 measures/offspring) sired by 215 fathers (1–51 measures/father). In this analysis, we analysed the birth interval length following from the birth of each grandchild. Consequently, grandchildren who were only-children or last born in their family were omitted from the analysis. Father effects on reproductive tenure length (figure 1*c*) were investigated for 564 offspring sired by 218 fathers (1–8 measures/father; sample sizes differ slightly because men who had one child had neither inter-birth intervals nor reproductive tenure lengths, and because birth intervals of greater than 10 years were excluded). All analyses control for confounding effects (if significant) of: maternal presence; birth cohort; ecology (coastal versus inland); socio-economic status (social class); and offspring sex and birth order (Lummaa *et al*. 1998; Lahdenperä *et al*. 2004*b*); as well as repeated measures of fathers. The analysis of age at first reproduction additionally controlled for father age (whether alive or dead), so that the result would not be confounded by the possibility that offspring who begin to breed early are more likely to have a younger, and hence more probably, living father. The analysis of inter-birth interval additionally controlled for early offspring survival (up to age 1) and repeated measures of offspring, while that of reproductive tenure lengths additionally controlled for offspring age at first reproduction.

### (b) Father presence and offspring fitness correlates

**Figure 2:**
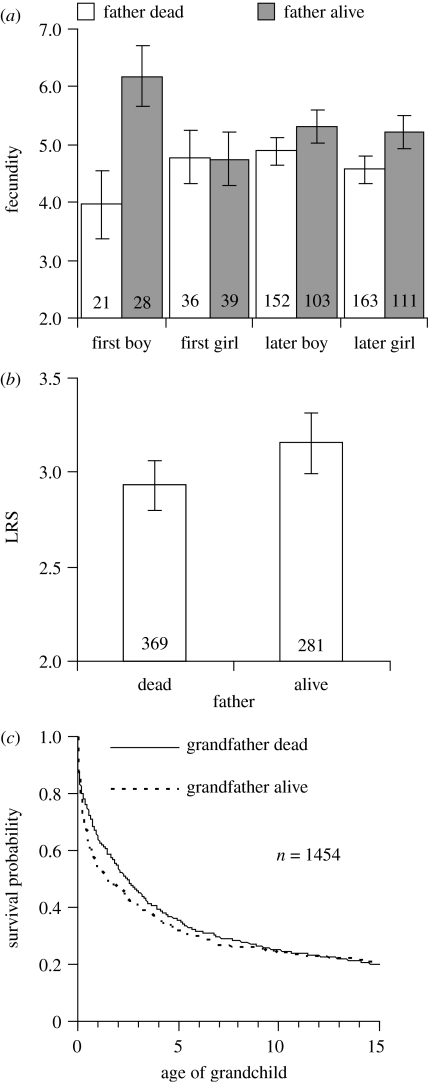
Father presence and fitness correlates of offspring and grandoffspring. A living father *(a)* increases his first-born son's lifetime number of children born (GLMM: interaction: *F*_1,637_=4.87, *p*=0.027), but has no effect on (*b*) the lifetime reproductive success of any of his children (GLMM: *F*_1,481_=1.45, *p*=0.23), presumably because (*c*) he has a non-significant tendency to have a negative effect on grandchild survival probability (log-rank test: *Χ*_1_^2^=2.50, *p*=0.11). Graphs (*a*,*b*) show predicted means (±1 s.e.) from GLMMs after controlling for significant effects outlined in the relevant section of the results and repeated measures of father (*p*_(*a*,*b*)_*=*0.15 and 0.0024, respectively).

Offspring fitness correlates were investigated in terms of lifetime fecundity (number of offspring delivered or sired in a lifetime) and lifetime reproductive success (number of children produced surviving to 15 years of age). Effects of father presence on these fitness correlates (figure 2*a*,*b*) were analysed using GLMM. We defined father presence/absence as whether the father was alive or dead at the time each offspring started reproducing. (Again, considering the number of years for which fathers were alive after this age failed to change the results: fecundity: GLMM, interaction between father presence in years, offspring sex and birth order, *F*_1,637_=3.40, *p*=0.06; and LRS: GLMM, father presence in years *F*_1,271_=1.71, *p*=0.19). Only individuals whose full life history was known with certainty were included, although the LRS analysis was weighted by the proportion of children in the offspring's family for which we also knew their survival status until age 15 with certainty. Both analyses were based on the productivity of 650 offspring sired by 229 fathers (1–8 measures/father). Both analyses control (where significant) for maternal presence, birth cohort, ecology (coastal versus inland), socio-economic status (social class) as well as offspring sex and birth order, and repeated measures of fathers.

In order to determine the mechanism through which any differences between lifetime fecundity and lifetime reproductive success might arise, we investigated the effects of having a living grandfather on grandchild survival probability to age 15 using a log-rank test (figure 2*c*). Survival curves were plotted using the Kaplan–Meier method. This analysis is based on the survivorship of 1454 grandoffspring in the presence/absence of 216 grandfathers at the time each child was born. Closer examination of grandfather effects on grandchild survival in age categories (0–2 years, 2–5 years, 5–15 years), using GLMM with binomial response term and logit link function and controlling for confounding terms and repeated measures of father/grandfather did not change our result of the log-rank test (GLMM results not shown).

### (c) Male longevity and fitness through grandfathering

**Figure 3:**
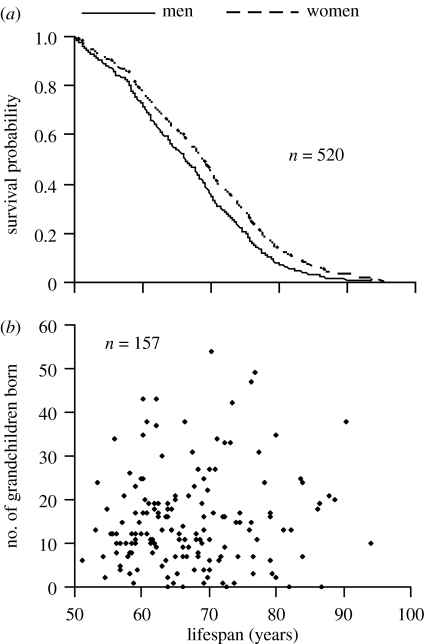
Male longevity and its effects on fitness. (*a*) Men have significantly lower survival probability after age 50 than women (Cox regression: *Χ*_1_^2^=6.59, *p*=0.01). (*b*) Male lifespan after age 50 is not associated with increased numbers of grandchildren born (fitness; GLM: *β*=0.079±0.090, *F*_1,153_=0.77, *p*=0.38). Graphs show predicted means after (*a*) controlling for confounding terms and (*b*) a scatter plot of the raw data to highlight the complete lack of relationship.

Sex differences in survival between men and women after age 50 (*n*=233 men, 288 women; figure 3*a*) were conducted using a Cox proportional hazards model in which birth cohort, ecology and socio-economic status were controlled. Individuals who survived beyond age 50 and were married only once during their lifetime were included in the model. Assumption of proportional hazards model was checked by including time-dependent covariates of explanatory variables in the model (Collett 2003). No evidence of non-proportionality of hazards was found.

To determine whether men gained fitness through grandfathering, we investigated the relationship between a male's longevity and the number of grandchildren ever ‘born’ to him irrespective of whether he was alive or dead (figure 3*b*) using general linear model (GLM), in which the number of grandchildren born was fitted as the response term to a normal error structure and male longevity was fitted as the main fixed effect. We restricted our data to include only those men who were married once during their lifetime and who lived past the age when 90% of men had finished reproducing (approx. 53 years; *n*=157). Our analysis controls for a man's wife's post-reproductive longevity, the number of children born to his wife during his reproductive years, birth cohort, ecology and socio-economic status.

## 3. Results

### (a) Father presence and offspring life-history traits

Reproductive careers started late in pre-industrial Finland, with men starting significantly later than women (mean men versus women: 28 (17–61) versus 26 (16–42) years: *t*-test; *t*_628_=4.74, *p*<0.0001). In the same populations of Finns, the presence of a mother is associated with a reduction in the age at first reproduction of all of her offspring by an average of 2.4 years (Lahdenperä *et al*. 2004*b*). Here, we show a similar result for fathers. After controlling for significant effects of maternal presence (*F*_1,502_=25.22, *p*<0.0001), birth cohort (*F*_4,447_=3.07, *p*=0.016), social class (*F*_2,653_=6.67, *p*=0.0014), individual sex (*F*_1,641_=9.23, *p*=0.0025) and father's age (*F*_1,540_=3.68, *p*=0.056), we found that all offspring in a family started reproducing at a significantly earlier age if their father was alive versus dead (figure 1*a*). The magnitude of this effect equates to a 2.3-year decrease in the age at first reproduction of offspring with a living father.

The mean interval between births in our population was 2.7 years (0.9–9.8 years). The presence of a mother is generally associated with a three-month reduction in inter-birth intervals by her offspring (Lahdenperä *et al*. 2004*b*). The effect of having a living father again is similar. After controlling for significant effects of the interaction between maternal presence and birth order (*F*_3,2387_=3.36, *p*=0.018), birth cohort (*F*_4,993_=3.20, *p*=0.013), ecology (*F*_1,361_=6.75, *p*=0.0098) and the survival of the previous child to 1 year (*F*_1,2445_=263.76, *p*<0.0001), we found that father presence had a significant effect on the birth intervals of his children (figure 1*b*). The magnitude of this father effect equates to a reduction in inter-birth interval of 2.6 months for all offspring with a living father.

The mean reproductive tenure length of offspring in our population was 11.1 years (0–45 years). In the presence of a living mother, offspring breed for an average 1.2 more years (Lahdenperä *et al*. 2004*b*). This was not the case in the presence of a living father. After controlling for significant effects of maternal presence (*F*_1,406_=6.17, *p*=0.013), ecology (*F*_1,185_=14.37, *p*=0.0002) and age at first reproduction (*F*_1,548_=69.40, *p*<0.0001), we found a significant three-way interaction (figure 1*c*). Father presence was only associated with an increase in the reproductive tenure lengths of his first-born offspring, but only if this was a son. In the presence of a living father, first-born sons bred for 3.5 years longer than first-born daughters and 2 years longer than later born offspring.

### (b) Father presence and offspring fitness correlates

Individual fitness will, in part, be the result of the number of children an individual produces in his/her lifetime (lifetime fecundity) and the number of offspring who survive to adulthood (lifetime reproductive success). On average, individuals had 4.7 offspring (0–16) in their lifetime and reared 3.1 (0–10) to adulthood. Offspring beginning to breed in the presence of their mother delivered 0.9 (18%) more children in their lifetime and reared 0.5 (16%) more to adulthood (Lahdenperä *et al*. 2004*b*). This was not the case for fathers. After controlling for significant effects of maternal presence (*F*_1,439_=8.04, *p*=0.0048), ecology (*F*_1,178_=35.09, *p*<0.0001) and social class (*F*_2,599_=14.09, *p*<0.0001), we found that fathers only increased the reproductive output of their first-born offspring if it was a son (figure 2*a*). Moreover, after controlling for significant effects of maternal presence (*F*_1,485_=4.43, *p*=0.036), ecology (*F*_1,183_=5.95, *p*=0.016) and social class (*F*_2,621_=15.56, *p*<0.0001), we found no evidence to suggest that father presence was associated with an increase in offspring lifetime reproductive success (figure 2*b*), irrespective of gender or birth order. One explanation for this lack of LRS effect is that the presence of grandfathers, unlike grandmothers (Lahdenperä *et al*. 2004*b*), tends to have a negative effect on the survival probability of grandchildren (log-rank test: *Χ*_1_^2^=2.50, *p*=0.11; figure 2*c*).

### (c) Male longevity and fitness through grandfathering

The survival probability of men versus women after the age of 50 years differed significantly, with men having a greater mortality risk than women (Cox proportional hazards model: *Χ*_1_^2^=7.07, *p*=0.0078, figure 3*a*). As a consequence, the average lifespan of a Finnish husband who survived to 50 years of age was significantly less than it was for their wife who survived to the age of 50 years (mean=66 (50–94) versus 69 (50–96) years; paired *t*-test; *t*_162_=3.10, *p*=0.0023). The mean number of grandchildren ever produced for men (who lived after age 50) was 15 (0–61). Mothers are known to gain two extra grandchildren for every 10 years they survive after the age of 50 years (up to age 75; Lahdenperä *et al*. 2004*b*). In contrast, after controlling for significant effects of ecology (*F*_1,153_=14.53, *p*=0.0002) and ‘fecundity’ during reproductive years (*F*_1,153_=24.29, *p*<0.0001), we found that the length of a man's lifespan (after age 50) failed to influence the number of grandchildren ever ‘born’ to him (figure 3*b*). Hence, men did not improve their evolutionary fitness by surviving beyond the reproductive capacity of their wife and helping to increase the reproductive output of his children in pre-industrial Finns.

## 4. Discussion

The long post-reproductive lifespan in women can be explained, in part, by fitness enhancing grandmother effects (see §1). We found that in pre-modern Finns, longer living men had some effects on the life history of their offspring, but these effects were insufficient to influence their fitness. More precisely, living fathers reduced the age at which offspring first reproduced and shortened inter-birth intervals, but had little effect on reproductive tenure lengths. Further, father influences on key life-history traits were largely insufficient to affect offspring fecundity and wholly insufficient to affect offspring lifetime reproductive success, presumably because he had no (or negative) effects on the survival of grandchildren. Consequently, although fathers had some significant influences on the underlying life-history traits of his offspring, these were insufficient to cause increments to fitness, and hence male lifespan beyond the menopause of his wife appears not to have been under positive directional selection in this monogamous society.

Previous investigations of (grand)father effects, with due regard to confounding sources of influence, have been conducted on only ten different human populations (table 1): two on contemporary Africans (hunter–gatherer and agro–pastoralist); two on largely contemporary farmers (Africa and West Indies) and six on largely historical farmers (Japan and Europe). In Finns, three key adult life-history traits that determine individual fitness are age at first reproduction, inter-birth interval and reproductive tenure length (Helle *et al*. 2005), with most of these traits having been shown to have a significant heritable basis (Pettay *et al*. 2005). Individuals who commence reproduction early, breed frequently and have a long period of reproductive tenure are most fit. Father effects on key life-history traits have been studied in five of the ten populations studied, but most of these consider only one trait. The evidence from these few studies show that father effects are typically absent, with only one study showing a significant effect; even then this effect was only common to maternal fathers. In rural Gambia, the presence of a maternal father is associated with a significant reduction in the age at which offspring first reproduce (Allal *et al*. 2004).

Previous studies have not investigated father effects on several underlying life-history traits in the same population. We found that the presence of a living father was associated with a lowering of age at first reproduction and a shortening of inter-birth intervals of all his offspring, and a lengthening of reproductive tenure lengths among first-born offspring (if they were sons). Fathers therefore appear to have the potential to benefit their own offspring in adulthood. Indeed, the father effects observed on age at first reproduction and inter-birth interval are comparable to mother effects from the same population (Lahdenperä *et al*. 2004*b*). One reason why our father results are stronger than those of previous studies is that our study population was strictly monogamous and we restricted our fathers to those who married only once during their lifetime. In other words, not only was the mating system of our study population likely to be conducive to fathers gaining fitness from helping their offspring to breed (Marlowe 2000), but also we restricted our analysis to the subset of fathers for whom grandfathering was the only way in which a man could gain fitness after the menopause of his wife. Hence, our study was deliberately set up to maximize the potential for father effects to be detected.

The question is, however, are such father effects sufficient to result in fitness pay-offs? Mother and father effects on offspring age at first reproduction and inter-birth intervals are comparable, but mothers have significant effects on the lifetime fecundity of all offspring (Lahdenperä *et al*. 2004*b*), while fathers only benefit first-born offspring (if male). In concordance, while mother presence is associated with an increase in the reproductive tenure lengths of all of her offspring, father presence is only associated with an increase in the reproductive tenure length of his first-born offspring (if male). That fathers only benefit their first-born sons is feasible. In historical Finland, the predominant household contained both parents and the family of one child (Moring 1993). Land transfers usually followed the male line and favoured the eldest, with the younger siblings moving out of the household in their early twenties, although they may return for brief periods until marriage. However, why mothers benefited all of the offspring similarly, while fathers only benefited a minority is currently unclear.

Nevertheless, offspring lifetime reproductive success was not enhanced by having a father present, and hence all father effects on offspring key life-history traits were insufficient to enhance offspring fitness. Furthermore, fathers failed to accrue fitness by remaining alive beyond the menopause of their wife and helping her. In the two other studies to investigate father effects on offspring lifetime reproductive success, our finding mirrors that found in a study of Dominicans (Quinlan & Flinn 2005), but contrasts with that of a study of Poles (Tymicki 2004). One reason why we failed to detect an effect on lifetime reproductive success is that having an alive grandfather was associated with a non-significant trend for grandoffspring to have reduced survivorship to adulthood. The reason for this is currently unclear, but one possibility is that old men dominated the shared food resource of the family over children or there existed some other resource conflicts in the family (Kemkes-Grottenthaler 2005). Paternal grandfather presence was also found to have a negative effect on the survival of grandchildren in historical Germany (Kemkes-Grottenthaler 2005) and of granddaughters in a study of pre-industrial Japanese (Jamison *et al*. 2002). Indeed, positive grandfather effects on grandoffspring survival are generally lacking. Of the eight studies to investigate effects on grandoffspring survival, only one has found a significant positive effect and this was for maternal grandfathers only (Beise 2005). The question remains though, why we and others (Quinlan & Flinn 2005) failed to detect an effect of fathers on offspring lifetime reproductive success, while the Polish study (Tymicki 2004) showed a significant positive effect. One possibility that cannot be ruled out is that because this Polish study was not restricted to pre-modern times (study period 1690–1968), the result might have been generated by increases in general survival rates following the demographic transition, leading to an accidental correlation between grandfather presence and grandchild survival.

In conclusion, our study is unique in having investigated grandfather effects on three underlying life-history traits known to influence fitness and in investigating the effect of the length of male lifespan (after age 50) on the total number of grandchildren born, which is one of the best long-term fitness measures currently available. The results of our study have at least three general and important implications. First, that we found significant effects of father presence on underlying offspring life-history traits but no effect on offspring fitness highlights the importance of measuring fitness before concluding whether statistically significant effects on individual life-history traits are biologically important. Second, that we have previously shown significant fitness consequences of grandmothering but failed to find a similar effect of grandfathering in the same population strongly suggests that the grandmother result is real and not a consequence of an unknown confounding variable. Finally, that we failed to find a significant grandfather effect in our monogamous society in which we restricted our data to those men who married only once in their lifetimes (and hence could only gain fitness by grandfathering after the menopause of their wife) strongly suggests that the evolution of prolonged life in men cannot be explained by the selective benefits of grandfathering. This counters accumulating evidence to suggest that mothers can gain significant increments to fitness by helping their offspring following menopause (see above). We suggest that if evidence for a grandfather hypothesis is lacking in a monogamous western society, then its general importance in shaping male lifespan during our more promiscuous (Dupanloup *et al*. 2003) evolutionary past is likely to be negligible. Long life in men must therefore arise through either the benefits of continued mating throughout life or as the unselected consequence of genes for longevity in females.

## Figures and Tables

**Table 1:** Effects of fathers/grandfathers on offspring key life-history traits, fertility and fitness correlates in human populations. (Studies shown are those published (including this study) having conducted statistical approaches which attempt to control for known potential confounds. All populations studied were farmers unless otherwise noted. AFR, age at first reproduction; IBI, inter-birth interval; RTL, reproductive tenure length; FEC, lifetime fecundity (i.e. number of born children); OFS, grandchild survival; LRS, lifetime reproductive success (i.e. number of children sired surviving to adulthood); FIT, number of born grandchildren; 0, non-significant; +, positive; −, negative. Note that negative effects on AFR and IBI are beneficial. Where two effects are shown, they refer to the effects of maternal/paternal (grand) fathers respectively, while one effect refers to overall (grand) father effects.)

population	AFR	IBI	RTL	FEC	OFS	LRS	FIT	reference
Ache hunter–gatherers of Paraguay (1890–1971)		0/0			0			Hill & Hurtado (1996)
Oromo agro–pastoralists of Ethiopia (1999–to date)					0/0			Gibson & Mace (2005)
Dominicans (1835–2004)	0^a^			0/0		0/0		Quinlan & Flinn (2005)
Gambians (1950–to date)	−/0				0/0			Sear *et al*. (2002) and Allal *et al*. (2004)
Japanese (1671–1871)					0/−b			Jamison *et al*. (2002)
Canadians (1680–1750)					+/0			Beise (2005)
Poles (1690–1968)				+/+		+/+		Tymicki (2004)
Germans (1720–1874)		0/0			0/0			Voland & Beise (2002)
Germans (1700–1899)					0/−			Kemkes-Grottenthaler (2005)
English (1770–1861)		0/0	0/0	0/0	0/0			Ragsdale (2004)
Finns (1719–1898)	−	−	+^c^	+^c^	0 (−)	0	0	this study

a Only the effect of woman's father considered.

b Negative effect on granddaughters only.

c Effects only on first-born sons.
